# Salicylic Acid Mixtures

**Published:** 1877-02

**Authors:** 


### SALICYLIC ACID MIXTURES.

Mr. A. Cassan recommends in the *Repertoire de Pharmacie* the following formula:

| | |
|---|---|
| Salicylic Acid | 1 drachm. |
| Citrate of Ammonia | 30 grains. |
| Brandy | 1 ounce. |
| Water | 5 ounces. |

The addition of citrate of ammonia is claimed to effect in no way the action of the acid, and to give no unpleasant taste to the solution. The citrate is made extemporaneously by saturating critric acid with ammonia.

